# Neuroprotective and Anti-Inflammatory Effects of *Rhus coriaria* Extract in a Mouse Model of Ischemic Optic Neuropathy

**DOI:** 10.3390/biomedicines6020048

**Published:** 2018-04-23

**Authors:** Saba Khalilpour, Ghazaleh Behnammanesh, Fouad Suede, Mohammed O. Ezzat, Jayadhisan Muniandy, Yasser Tabana, Mohamed B. K. Ahamed, Ali Tamayol, Amin Malik Shah Majid, Enrico Sangiovanni, Mario Dell’Agli, Aman Shah Majid

**Affiliations:** 1 Department of Pharmacological and Biomolecular Sciences (DiSFeB), Università degli Studi di Milano, Via Balzaretti, 9-20133 Milan, Italy; enrico.sangiovanni@unimi.it (E.S.); mario.dellagli@unimi.it (M.D.); 2 Department of Medical Pharmacology and Physiology, University of Missouri, Columbia, MO 65212, USA; gb3nc@mail.missouri.edu; 3 EMAN Biodiscoveries Sdn. Bhd., Suite 126, Level 1, EUREKA Complex, University of Science Malaysia (USM), Minden Gelugor 11800, Penang, Malaysia; fouad@emanbio.com(F.S.); tabana@ualberta.ca (Y.T.); khadeer@emanbio.com (M.B.K.A.); aminmalikshah@usm.my (A.M.S.M.); 4 School of Pharmaceutical Sciences, University of Science Malaysia, Minden Gelugor 11800, Penang, Malaysia; edw.mohamed_oday@uoanbar.edu.iq (M.O.E.); jaegan_87@hotmail.com (J.M.); 5 Faculty of Pharmacy and Pharmaceutical Sciences, University of Alberta, Edmonton, AB T6G 2R3, Canada; 6 Department of Mechanical and Materials Engineering, University of Nebraska-Lincoln, Lincol, NE 68508, USA; atamayol@unl.edu; 7 John Curtin School of Medical Research, Australian National University, Canberra 2601, Australia; 8 Centre for Natural Product and Angiogenesis Research/Department of Pharmacology, Faculty of Medicine, Quest International University, Ipoh 30250, Malaysia

**Keywords:** neuroprotection, *Rhus coriaria*, linoleic acid, ischemic optic neuropathy, anti-neuroinflammation

## Abstract

Modulating oxidative stresses and inflammation can potentially prevent or alleviate the pathological conditions of diseases associated with the nervous system, including ischemic optic neuropathy. In this study we evaluated the anti-neuroinflammatory and neuroprotective activities of *Rhus coriaria* (*R. coriaria)* extract in vivo. The half maximal inhibitory concentration (IC_50_) for DPPH, ABTS and β–carotene were 6.79 ± 0.009 µg/mL, 10.94 ± 0.09 µg/mL, and 6.25 ± 0.06 µg/mL, respectively. Retinal ischemia was induced by optic nerve crush injury in albino Balb/c mice. The anti-inflammatory activity of ethanolic extract of *R. coriaria* (ERC) and linoleic acid (LA) on ocular ischemia was monitored using Fluorescence Molecular Tomography (FMT). Following optic nerve crush injury, the mice treated with 400 mg/kg of ERC and LA exhibited an 84.87% and 86.71% reduction of fluorescent signal (cathepsin activity) respectively. The results of this study provide strong scientific evidence for the neuroprotective activity of the ERC, identifying LA as one of the main components responsible for the effect. ERC may be useful and worthy of further development for its adjunctive utilization in the treatment of optic neuropathy.

## 1. Introduction

Optic neuropathy is a neurodegenerative disorder that involves damage to the optic nerve, which can be caused by acute or intermittent insults leading to visual dysfunction. Axonal degeneration of optic nerve and loss of retinal ganglion cells are the main factors responsible for this disorder [1]. Ischemic optic neuropathy (ION) is the most common type of acute optic neuropathy in older individuals and is caused by inadequate blood supply (ischemia) to the optic nerve [2,3]. The prevalence of ION in individuals over 50 years of age is 2–10 per 100,000 individuals [3].

The specific pathophysiology of ION has not yet been identified, but it is related to weak blood flow throughout the optic nerve head. Ischemia-reperfusion injury refers to any clinical condition involving inadequate oxygen consumption, such as stroke, trauma, shock, cerebral ischemia, or ischemic optic neuropathy [4]. Initially, the similar reduction in blood flow in brain ischemia results in a low concentration of oxygen and glucose. The resulting hypoxic conditions lead to repletion of lactate through anaerobic glycolysis and generation of oxygen free radicals which contribute to cellular apoptosis. Oxidative stress is believed to play a major part in the pathogenesis of optic neuropathy [1]. Apoptosis, or programmed cell death, is a method of cell loss during ischemia [5].

Free radicals results in upregulation of pro-inflammatory factors such as interleukins, platelet activating factor, and tumor necrosis factor α [1]. The major factors involved in the molecular mechanism of inflammation in neurodegenerative diseases like optic nerve degeneration are cytokins, mitogen-activated protein kinase, nuclear factor kappa B (NF-κB), cyclooxygenases, lipoxygen, nitric oxide synthase, and cathepsins [1]. Cathepsins constitute a family of proteases and inflammatory response mediators that are associated with regulation of a type of cell death called caspase-dependent cell apoptosis [2,3]. They have been shown to be a key initiator of apoptosis by directly activating caspases [5,6]. Induction of an inflammatory response by reperfusion following ischemic tissue injury may increase the intensity of damage [7].

There are several anti-inflammatory agents with neuroprotective effects, including aspirin (acetylsalicylic acid (ASA)), the hydrogen sulfide-releasing derivatives of aspirin (ACS14 (2-acetyloxybenzoic acid 4-(3-thioxo-3*H*-1,2-dithiol-5-yl)phenyl ester)), and a latanoprost acid derivative (ACS67) [1,8]. There is also evidence that supports the neuroprotective properties of sulfur compounds [9]. In recent years, several biological activities of polyphenols such as genistein have been described as preventing the progression of cancer, cardiovascular diseases, diabetes, neurodegenerative diseases, obesity, and aging [10,11]. Antioxidants and especially plant-derived antioxidants can play a role in the control or deceleration of the progression of neurodegenerative disorders such as optic neuropathies, glaucoma, Alzheimer’s disease, Parkinson’s disease, and ischemic or hemorrhagic stroke [1,12].

*Rhus coriaria* (*R. coriaria)*, a medicinal plant belonging to the Anacardiaceae family, is widely grown throughout the Mediterranean region. Historically, leaves and berries of *R. coriaria* were known to have remarkable medicinal value in Middle Eastern herbal medicine [12]. Recent phytochemical studies of berries of *R. coriaria* have proved the presence of various antioxidants including phenolics and fatty acids [13]. Linoleic, gallic, oleic, palmitic, and stearic acids are the major components that have been identified in the fruits of this herb in previous studies [12]. The role of *R. coriaria* as antidiarrheal, anti-indigestion, anti-anorexia, antihemorrhagic, hyperglycemic, atheroprotective and neuroprotective, properties have been previously assessed by in vitro and in vivo studies [5,12]. In the present study, we evaluated the neuroprotective action of *R. coriaria* in an in vivo model of ION. The focus of this research was to determine the role of *R. coriaria* on the factors involved in optic nerve degeneration including oxidative stress, neuroinflammation, and cathepsin activity.

## 2. Experimental Section

### 2.1. Collection and Authentication of the Herb

*R. coriaria* samples were collected in the Taleghan region located 120 km northwest of Tehran, Iran. The plant material was authenticated by a botanist, Dr. Rahmad Zakaria and submitted at Herbarium Unit, School of Biological Sciences, University of Science Malaysia, where a voucher specimen was deposited (reference# 11526).

### 2.2. Preparation of R. coriaria Extract

The collected fruits were cleaned and dried at 35 °C for 24 h. To prepare the extract by the maceration process, powder form of the dried herb was added to ethanol and subjected to maceration [5]. The mixture of herb and solvent was placed in a shaker for 48 h at room temperature to be mixed gently using a magnetic stirrer. After cooling, the obtained solution was filtered using Whatman filter paper and concentrated using a rotary evaporator (RE121 Buchi, New Castle, DE, USA) under vacuum. The solvent was evaporated and removed, and the remaining material was freeze-dried (Labconco™, Kansas City, MO, USA). The obtained extract was kept in sterile glass containers at 2 °C for further use.

### 2.3. Antioxidant Capacity Assays

#### 2.3.1. ABTS (2,2′-Azino-*Bis*(3-Ethylbenzothiazoline-6-Sulphonic Acid) Radical Scavenging Assay

The total antioxidant capacity was assessed using a decolorization method described previously [14]. ABTS radical cation (ABTS^+^) solution was prepared by mixing 7 mM ABTS with deionised water. Fresh radicals were prepared by mixing 2.5 mL of 2.45 mM potassium persulfate with 2.5 mL of 7 mM ABTS aqueous solution. Stock solutions of the test extract (ethanolic extract of *R. coriaria* (ERC)) and standard (ascorbic acid) were prepared in methanol. The tested extract (0.1 mL) at different concentrations was added to 0.9 mL ABTS^+^ solution, and the absorbance was measured at 734 nm after 6 min. For the negative control, 0.9 mL of ABTS^+^ was mixed with 0.1 mL of methanol. All tests were performed in triplicate.

#### 2.3.2. DPPH (2-Diphenyl-1-Picrylhydrazyl) Scavenging Assay

The assay was performed using a previously described method [15]. Stock solution of the ERC was prepared in methanol and diluted to different concentrations in the range of 1 to 30 µg/mL. In a test tube, 2.5 mL of different concentrations of test samples dissolved in MeOH:H_2_O (1:1) were added to 1 mL of 0.3 mM DPPH prepared in methanol. For the negative control, 2.5 mL of methanol were mixed with 1 mL of 0.3 mM DPPH. The mixture was reacted at 25 ± 2 °C for 30 min, after which the absorbance was measured at 517 nm using the microplate reader (TECAN Infinite Pro^®^ M200, Männedorf, Switzerland), and the amount of remaining DPPH was determined. The experiment was performed in triplicate.

#### 2.3.3. B-Carotene Bleaching Assay

The β-carotene/linoleic acid (LA) emulsion was used to measure the β-carotene oxidation and free radical scavenging action of the extract [16]. First, 2 mg of β-carotene were dissolved in 10 mL of chloroform. Then, 1 mL of β-carotene solution was mixed with 20 mg of purified LA and 200 mg of Tween 40 emulsifier in a round-bottom flask. Rotary vacuum evaporator was used to remove chloroform. Distilled water (50 mL) was added to the flask and the mixture was stirred with a vortexer. Aliquots of this emulsion were mixed with different concentrations of the ERC. Butylated hydroxytoluene (BHT) was used as the reference standard. The samples were shaken and incubated at 50 °C in a water bath. The zero time absorbance was assessed at 470 nm using a microplate reader and recorded at 20 min intervals. The assays were performed in triplicate.

### 2.4. Characterization of the Extract

#### 2.4.1. Estimation of Total Phenolic Content

The total phenolic content of the extract was determined using a colorimetric assay. Gallic acid was used as the standard reference. A series of concentrations of gallic acid (1 to 30 μg/mL) was prepared in distilled water, and 1 mL of each concentration was mixed with 0.5 mL of Folin-Ciocalteu reagent. The test tube was kept for 4 min at room temperature (25 ± 2 °C) after shaking. The sample was mixed with 1 mL of 20% (*w*/*v*) solution of Na_2_CO_3_ and 6 mL of distilled water, shaken, and kept in a dark room for 2 h at 25 ± 2 °C. The absorbance was read at 765 nm using the microplate reader. A stock solution of 1 mg/mL of the ERC was prepared in methanol and diluted, obtaining different concentrations (1, 5, 10, 20 and 30 μg/mL). The results are expressed as µg gallic acid equivalents per gram of the extract (µg GAE/g extract). The results of three experiments are presented as mean ± SEM (standard error).

#### 2.4.2. Total Flavonoids Assay

Quercetin was used as the standard reference in the colorimetric method used to determine total flavonoid content [17]. A stock solution of 2 mg/mL of the ERC and 1 mg/mL of the standard was prepared in methanol. The volume of 1 mL of each concentration of the standard solution (1–30 µg/mL in distilled water) and the test extract solution were mixed with 4 mL of distilled water and 0.3 mL of 5% sodium nitrite solution (NaNO_2_), respectively. After standing for 5 min at 25 ± 2 °C, 0.3 mL of aluminum chloride (10% solution) was added to the mixture in a 10 mL volumetric flask. Next, 2 mL of 1 M NaOH were added and the total volume was increased to 10 mL by adding distilled water. The absorbance was read at 510 nm using the microplate reader. The results are expressed as µg quercetin equivalents per gram of the extract (µg QE/g extract). The results of three experiments are reported as mean ± SEM.

#### 2.4.3. Gas Chromatography-Mass Spectrometry (GC-MS) Screening

GC-MS analysis is used to evaluate the various phytoconstituents obtained from different extraction procedure [18]. An Agilent GC-MS (6890N/5973I) coupled with electrospray ionization and a single quadrupole detector was used to separate the various phytoconstituents using an HP-5 MS capillary column (0.25 mm × 30 m × 0.25 µm film thickness). The temperature of oven was retained at 70 °C for 2 min and enhanced at 20 °C/min to 305 °C and retained there for 1 min. Helium gas with a flow rate of 1.2 mL/min was used as the carrier gas, and 1 µL of the sample was injected into the GC-MS for analysis. The MS electrospray ion source operated at 70 eV, and the acquisition range was between 35 and 700 *m*/*z* with a scan rate of 1 scan/s. The presence of chemical constituents in the extract was confirmed by referring to the NIST (national institute of standards) library of the system and by their retention indexes.

### 2.5. In Vivo Study of the Mouse Ischemic Optic Neuropathy Model

#### 2.5.1. Animal Studies

The Animal Ethics Committee of the University of Science Malaysia approved the study involving mice. The animals were obtained from the Animal House Unit, University of Science Malaysia. In handling the animals, all procedures were carried out following the Animal Ethics Guidelines of University of Science Malaysia (USM/Animal Ethics Approval/2013/(90) (543), 11/12/2013). Grouping and numbers of mice were as follows: 15 male mice, aged 8–12 weeks, were divided into five groups (*n* = 3) for positive control, negative control, and three groups of test animals).

#### 2.5.2. Animal Treatment

The three test groups of animals were administered orally with: (i) 200, (ii) 400 mg/kg body weight of the ERC, and (iii) 400 mg/kg of LA, once a day for 10 days before performing an optic nerve crush injury. The two control groups received just the normal diet.

#### 2.5.3. Optic Nerve Crush Injury

An optic nerve ischemia procedure was performed on the negative control group and the three treated groups after anesthetizing the mice with ketamine (90 mg/kg body weight) and xylazine (10 mg/kg body weight) via intraperitoneal injection. In the left eyes of animals, the ocular muscles around the eyeball and optic nerve were held apart with forceps. Then the 10-0 surgical suture was used to make crush lesions. One end of suture was kept near the optic nerve with forceps and the other end was pulled with a weight to constrict it for 60 s and then it was released [19]. In this way the retinal ischemia was induced by interruption of the blood flow through the ophthalmic vessels. The animals subjected to ischemia were kept in normal condition in animal house.

#### 2.5.4. Fluorescence Molecular Tomography (FMT) Imaging

The FMT 2000 (Fluorescence Tomography System, PerkinElmer, Waltham, MA, USA) was used for this study. Preparation for FMT imaging began 24 h after optic nerve injury and induction of ocular ischemia. Animals in all five groups were injected intravenously via a tail vein with 1 nmol ProSense 750 EX (VisEn Medical, Bedford, MA, USA) in 200 μL of saline. Twenty-four hours after the ProSense injection, they were anesthetized and imaged by FMT. This system was able to detect fluorescence and resolve the probe signal in the targeted region. The mouse was placed horizontally on a fixed stage at a known distance from a camera. For acquisition of the data sets a fluid was added to the imaging chamber and then the data reconstructions were run on the system within 24 h. The three-dimensional regions of interest (ROI) were selected using the software supplied with the instrument (WinLight32, Berthold Technologies, Bad Wildbad, Germany). The ROI data were collected and exported to Microsoft Excel for data analysis [20].

### 2.6. Statistical Analysis

Statistical analyses were carried out with Microsoft Excel and Sigma Plot software version 13.0. Comparisons between test and control groups were made using *t*-tests. Data were expressed as a mean percentage of the control plus SEM, and *p*-value < 0.05 was considered to be significant.

## 3. Results

### 3.1. ABTS Radical Scavenging Assay

**Figure 1:**
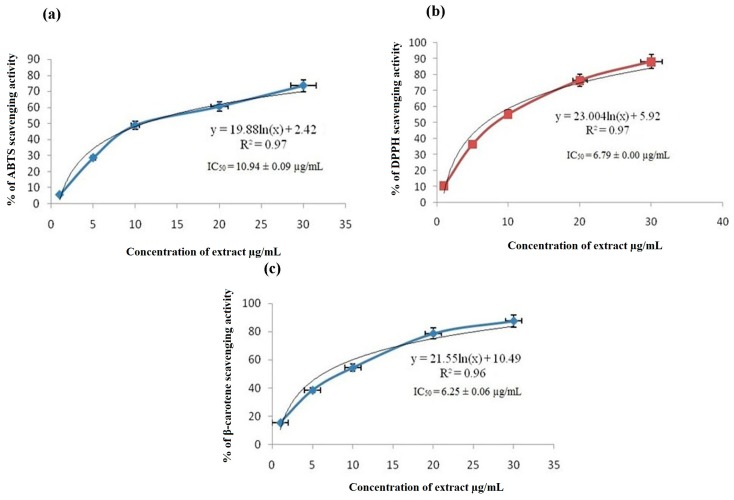

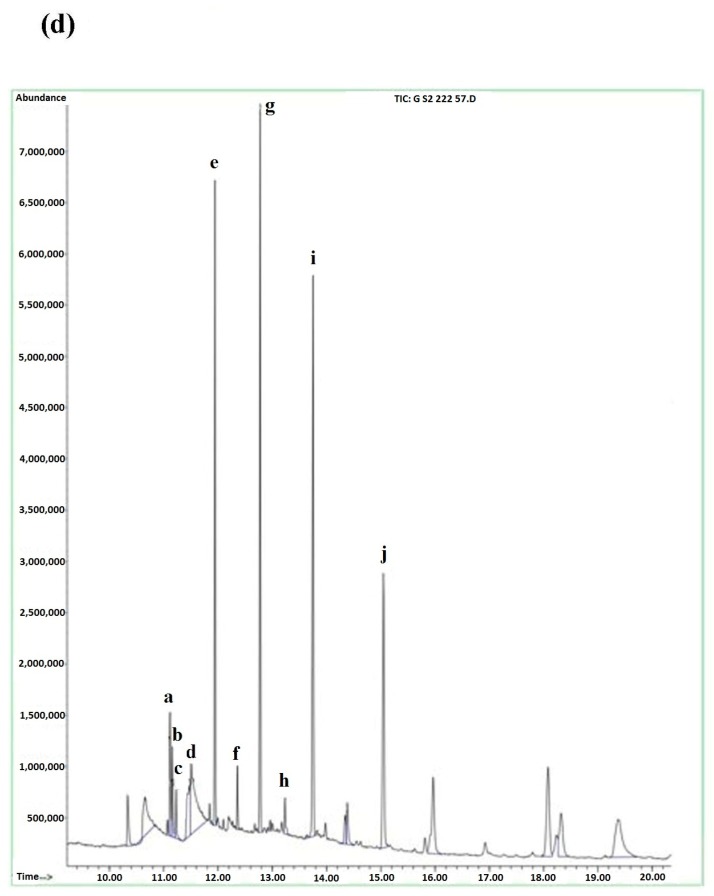
(**a**) Free radical scavenging activity of the ethanol extract of *R. coriaria* measured by ABTS assay. Results are expressed as mean ± SEM (*n* = 3); (**b**) effects of the *R. coriaria* ethanol extract on DPPH scavenging activity; (**c**) results of the β-carotene bleaching assay of the *R. coriaria* ethanol extract; (**d**) GC-MS chromatogram of the *R. coriaria* ethanol extract.

The ERC had effective free radical scavenging ability (Figure 1a), as indicated by the IC_50_ value of 10.94 ± 0.09 µg/mL (*n* = 3, *p*-value < 0.001). The IC_50_ of the standard (ascorbic acid) was 4.97 ± 0.056 µg/mL. The ability of the ERC to scavenge ABTS radical (ABTS^+^) was noted to be very significant compared to the control. The ability of ERC to quench ABTS^+^ is an evidence for its action in preventing oxidative damages caused by free radicals.

### 3.2. DPPH Scavenging Assay

Figure 1b shows the concentration-response curve of the effect of the ERC on DPPH scavenging activity. The percentage of DPPH scavenging activity of the extract is represented as mean ± SEM. Serial dilution of the extract concentration to 1, 5, 10, 20, and 30 µg/mL was used throughout the experiment. The results showed that the extract possessed potent scavenging ability, as it reduced the purple DPPH reagent to yellow by donating its hydrogen. The extract quenched DPPH in a concentration-dependent manner. The IC_50_ of DPPH scavenging activity was 6.79 ± 0.009 µg/mL (*n* = 3, *p*-value < 0.05), whereas that of the standard Rutin used in clinical practice was 3.23 ± 0.019 µg/mL.

### 3.3. β-Carotene Bleaching Assay

Figure 1c shows the results of the β-carotene bleaching activity. The low IC_50_ value of 6.25 ± 0.06 µg/mL (*n* = 3, *p*-value < 0.05) shows that the ERC inhibited β-carotene bleaching and that it had high radical scavenging activity. BHT was used as the standard, and its IC_50_ was 0.89 ± 0.039 µg/mL. This result demonstrates that ERC can act as a hydrogen-donating antioxidant to slow down the oxidation and β-carotene bleaching. A 50% reduction of the yellowish color of β-carotene by the extract was seen at 6.25 ± 0.06 µg/mL.

### 3.4. Assays for Total Phenolics and Total Flavonoids Content

In an effort to reveal the chemical components that give rise to the antioxidant activities of the ERC, phytochemical screening was undertaken, including an assessment of total phenolics and flavonoids. Table 1 summarizes the amounts of total flavonoids and phenolics present in the ERC extract. The total phenolic content was higher (9353 ± 83 µg GAE/g) than the total flavonoid content (1393 ± 32 µg QE/g) (Table 1). Phenolic compounds can play a major role in the prevention of various pathological conditions, including neurodegenerative diseases, by neutralizing chemically active oxidants [1]. It can thus be argued that the antioxidant activity of the ERC correlates directly with the demonstrated presence of phenolics and flavonoids.

### 3.5. GC-MS

The GC-MS method employed in this study was performed to further characterize for the chemical constituents present in the extract (Figure 1d). The presence of chemical components was confirmed by referring to the national institute of standards and technology (NIST) library of the system and by the retention indexes. In general, the GC-MS method applied was functional in screening unknown plant extracts. The chemical compounds found in the extract are listed in Table 2. LA (9,12-octadecadienoic acid (Z,Z)-) (12.39%), γ-sitosterol (11.95%), campesterol (9.32%), stigmasta-5,24(28)-dien-3-ol (8.95%), and octadecane (7.85%) were the major constituents.

### 3.6. FMT Imaging

**Figure 2:**
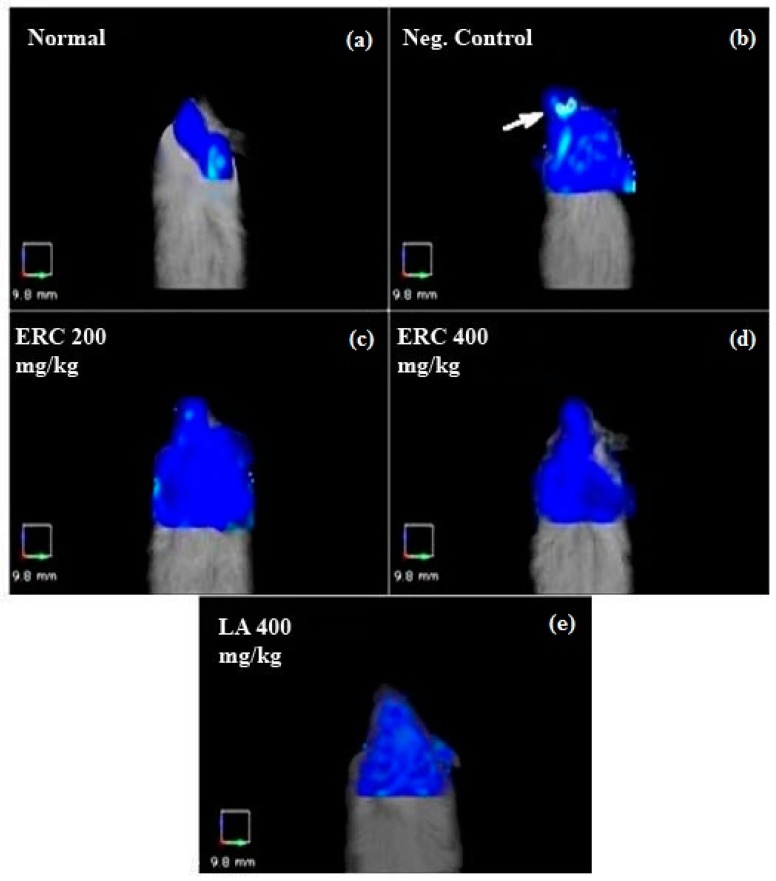
Fluorescence molecular tomography images without the region of interest (ROI). In this figure, the ROI area was not selected, but the differences in fluorescence intensities between the negative control (**b**) and the treated groups (**c**–**e**) is obvious. The negative control was the animal with induced ischemia at the left optic nerve/eye. The untreated normal animal was used as the positive/normal control (**a**). ProSens 750 was used as the inflammatory (cathepsin) fluorescent probe. In the group treated with 400 mg/kg body weight of ERC (**d**) and LA (**e**), the cathepsin fluorescence signal was significantly lower than that of the negative control.

**Figure 3:**
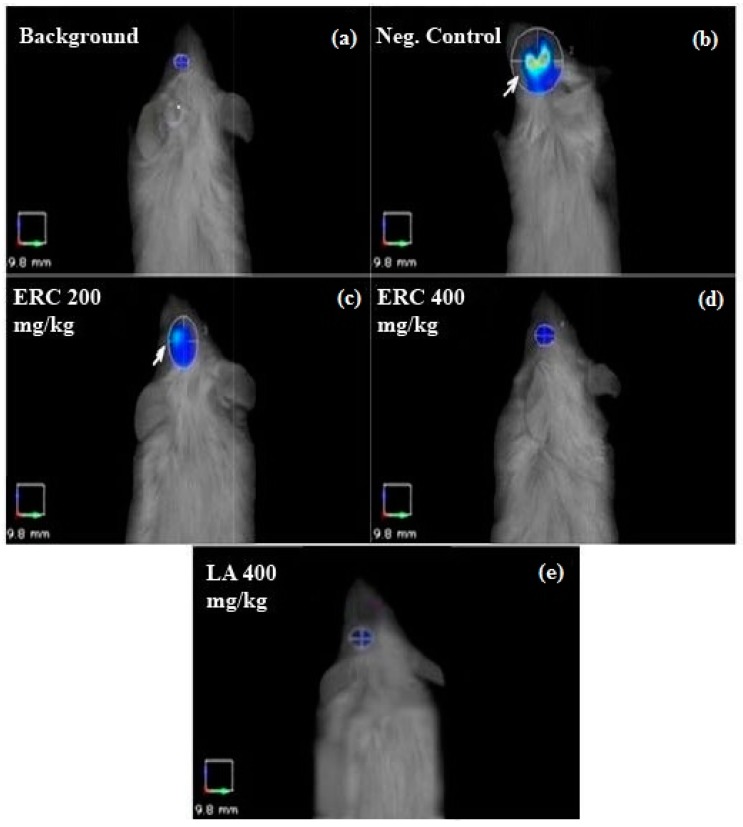
Fluorescence molecular tomography images with the region of interest (ROI). The ROI proximal to the eye area was selected to focus on the ischemic eye. In the negative control (**b**), the fluorescence signal was very intense (white arrow), whereas, in the treated ischemic eyes, treatment with LA (**e**) and different concentrations of ERC (**c**,**d**) reduced the fluorescence signal of the ROI. The image of the normal/positive control was used as the background.

**Figure 4:**
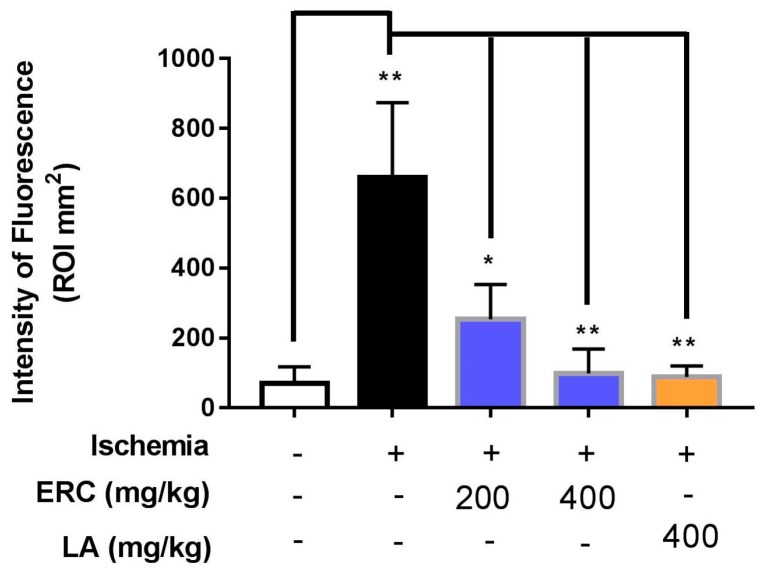
Graphical representation of the intensity of the fluorescence signal generated after induction of ischemia. Animals treated with ERC and LA exhibited a significant reduction in the ischemia-induced signal compared to the untreated group (control). Data are expressed as mean ± SEM (*n* = 3), * *p*-value < 0.05, ** *p*-value < 0.01.

Imaging by three-dimensional FMT was performed to quantify the inflammatory molecular response at the site of ischemic injury in the injured optic nerve and the protective role of ERC and LA (Figure 2). In our experiment, none of the animals in the five groups died during the experiments. After imaging, the images and data sets were analyzed before and after selecting the ROI. To measure the induced optic nerve ischemia, the images were focused on anatomical positions specific to the eye (Figure 3). There was no increase in fluorescence signal from the normal control group (Figure 3 and Figure 4), which indicated that pan-cathepsins were not being activated in the normal mouse optic nerve.

Data analysis revealed significant differences between in vivo FMT signal intensities of treated groups and the negative control, even before ROI selection (Figure 2). The reductions in fluorescence volume in the ROI for the group treated once a day for 10 days with 200 and 400 mg/kg of the ERC were exported and measured (Figure 4). The average fluorescence intensity within the ROI of the left eye after induction of ischemia for the negative control was 659.32 ± 123.57 mm^2^, for the group treated with 200 mg/kg of the ERC it was 253.56 ± 57.64 mm^2^, and for the group treated with 400 mg/kg of the ERC it was 99.30 ± 40.28 mm^2^. In contrast to the negative control, 61.54% and 84.87% inhibition of ischemia were exhibited in the groups treated with 200 and 400 mg/kg of the ERC, respectively (Table 3). For the group treated with 400 mg/kg of LA, the average fluorescence intensity after induction of ischemia was 87.20 ± 50.12 mm^2^. LA exhibited 86.71% inhibition of ischemia in comparison with the negative control.

For the ischemia-induced group (negative control), FMT signals recorded high fluorescence intensities of 659.32 ± 123.57 mm^2^. The high fluorescence signal in the damaged area was in response to activation of the probe by binding to cathepsin. This is indicative of initiation and promotion of neural damage. In the mice treated with 200 and 400 mg/kg of the ERC, the signal intensities of this fluorescent probe were significantly lower (253.56 ± 57.64 mm^2^ and 99.3 ± 40.28 mm^2^, respectively; Figure 4), which supports the in vivo neuroprotective efficacy of the ERC.

## 4. Discussion

The present study explored the neuroprotective potential of *R. coriaria* fruit extract using an array of studies to define the characteristic features and the biological target(s) that contributed to neuroprotection in an in vivo model of ION. Alcohols are frequently used as solvents to extract various bioactive ingredients such as phenolics and flavonoids [21]. The antioxidant property of the ethanolic extract of *R. coriaria* (ERC) was tested using several assays.

The identification and characterization of major components of *R. coriaria* fruit extract has been performed in previous studies [22,23]. In this study, GC-MS analysis was performed to screen the major constituent(s) of the ERC that are responsible for its neuroprotective activity. The GC-MS method applied in this study proved useful in screening unknown components in the extract tested as well as providing a view of the existence of an abundance of polar compounds. The major fatty acids found in the extract were derivatives of the polar fatty acid including LA, which is in agreement with literature [24]. LA constituted 12.39% of the total content of the extract and has been shown to possess neuroprotective and antioxidant activities. γ-sitosterol was present at 11.95%, which is also a common chemical constituent of several neuroprotective herbs such as *Vitis vinifera, Ginkgo biloba*, and *Bacopa monnieri* [25]. Campesterol and stigmasta-5,24(28)-dien-3-ol were present at 9.32% and 8.95%, respectively. They have also been detected in other herbal extracts with potent neuroprotective, antioxidant, and anti-inflammatory activities [26].

To assess the effects of the ERC on recovery after ischemia, different concentrations of the extract were tested for protective effects against ischemia in a mouse ION model; for details please refer to methods section. Studies of retinal ischemia can be simulated using a number of in vivo models. The albino Balb/c mouse, for example, is easy to handle and has a suitable retinal vasculature construction with similarities to humans. The goal of optic nerve crush method to induce ischemia caused by reduced blood supply to axons, leading to axonal damage [27]. We divided the animals into five groups: (i) positive control not injured without receiving any treatment; (ii) negative control injured without receiving any treatment; (iii) injured and treated with 200 mg/kg body weight of ERC; (iv) injured and treated with 400 mg/kg body weight of ERC; and (v) injured and treated with 400 mg/kg body weight of LA once a day for 10 days. The imaging probe used in this experiment was the pan-cathepsin fluorescent ProSense 750, which can be activated by cathepsins B, L, S, K, V, and D and turn into a highly fluorescence signal [28]. Cathepsins act as mediators of lysosomal proteases in apoptosis processes. Reactive oxygen species-dependent activity on cathepsin stimulation has also been demonstrated in studies of caspase-dependent apoptosis [29].

The significant antioxidant and the inhibition of the inflammatory mediator cathepsin by the ERC in response to neuronal ischemic injury provide supporting evidence of the neuroprotective property of the ERC. The high percentage of anti-inflammatory activity exhibited by the ERC in this study was perhaps due to the high content of phenolic compounds and the chemical constituents screened by GC-MS (Table 2). On the other hand, our results showed that, at the concentration of 400 mg/kg, LA was more effective (86.71%) than ERC (84.87%) at inhibiting ischemia, but there was no significant difference between them. We can, therefore, suggest that LA, a major component in ERC, may contribute significantly to the observed pharmacological activity. In support of the present findings, extensive studies have demonstrated the neuroprotective effect of LA and phenolic compounds in neurodegenerative disease models [30].

## 5. Conclusions

In this study, the ethanol extract of *R. coriaria* was prepared and evaluated for its neuroprotective effects in vivo. The antioxidant capacity of the ERC was demonstrated by different assays, including DPPH, ABTS, and β-carotene bleaching tests. In the central nervous system, a stroke occurs as a result of the reduction or blockage of blood flow in the affected region, leading to ischemic damage of neurons and other cells. In the present study, the interruption of blood circulation and induced axonal lesion to the optic nerve axon in the mouse model of ischemic optic neuropathy was carried out as a proof of the concept. In chemical screening studies of the extract, GC-MS and phytochemical analysis were used to reveal the presence of secondary metabolites (phenolics and flavonoids). The active compounds reported in the extract were LA, γ-sitosterol, campesterol, and stigmasta-5,24(28)-dien-3-ol, which have been previously identified as active constituents of herbal extracts with antioxidant, anti-inflammatory and neuroprotective properties [25].

Using the optic nerve crush method, the extract was found to inhibit the inflammatory response to ischemia-reperfusion in vivo by inhibiting the activity of the inflammatory mediator cathepsin. One of its major components, LA exhibited a comparable inhibitory effect on ischemia. LA may play an important role in its anti-inflammatory potential, and this mechanism may be partly responsible for its efficacy. The results of this study provide scientific evidence for the neuroprotective activity of the ERC. It is plausible, therefore, to conclude that the ERC may be useful and worthy of further development for its adjunctive use in the treatment of neurodegenerative diseases including ischemic optic neuropathy. Nevertheless, further toxicological studies must be carried out to evaluate its safety and potential hazards.

## Figures and Tables

**Table 1:** Phytochemical contents of the *R. coriaria* ethanol extract (ERC). The data are means ± SEM (*n* = 3).

Test Sample	Total Phenolics (µg GAE/g)	Total Flavonoids (µg QE/g)
ERC	9353 ± 83	1393 ± 32

**Table 2:** GC-MS screening of the phytochemicals present in the *R. coriaria* ethanol extract.

Peak	Compound	Ref	Quality	Peak Height	R.T. Min	% Area	RI
a	Tricosane	114376	99	1,192,748	11.118	2.65%	1408.8
b	Heptacosane	113306	99	826,548	11.16	1.87%	1496.4
c	Nonacosane	116666	98	451,294	11.229	1.03%	1632.8
d	Octadecane	106289	95	680,873	11.514	7.85%	2195
e	Campesterol	130016	97	5,389,423	11.944	9.32%	2298.8
f	8,11-Octadecadienoic acid, methyl ester	136481	97	607,878	12.361	1.10%	2398.4
g	γ-Sitosterol	136487	98	6,507,080	12.777	11.95%	2497.9
h	9,12,15-Octadecatrienoic acid, methyl ester	91035	96	331,695	13.235	0.90%	2599.5
i	Linoleicacid (LA) (9,12-Octadecadienoic acid (Z,Z)-)	151555	99	5,171,060	13.756	12.39%	2697.9
j	Stigmasta-5,24(28)-dien-3-ol	158131	99	2,634,496	15.054	8.95%	2945.6

**Table 3:** Comparison of fluorescence intensity (mm^2^) and % of ischemia inhibition in treated and control groups.

Test	Fluorescenceintensity (mm^2^) (Mean)	SEM	% Inhibition Ofischemia
Normal control	69.66	48.56	—
Ischemia (negative control)	659.32	123.57	—
ERC 200 mg/kg	253.56	57.64	61.54
ERC 400 mg/kg	99.30	40.28	84.87
LA 400 mg/kg	87.20	50.12	86.71
